# CD34^+^VEGFR-3^+^ progenitor cells have a potential to differentiate towards lymphatic endothelial cells

**DOI:** 10.1111/jcmm.12233

**Published:** 2014-01-22

**Authors:** Yu-zhen Tan, Hai-jie Wang, Mei-hua Zhang, Zhe Quan, Ting Li, Qi-zhi He

**Affiliations:** a Department of Anatomy, Histology and Embryology, Shanghai Medical School of Fudan UniversityShanghai, China; b Shandong Provincial Key Laboratory for Improving Birth Outcome TechniqueJinan, China; c Shanghai First Maternity and Infant Hospital, Tongji UniversityShanghai, China

**Keywords:** Endothelial progenitor cells, VEGFR-3, VEGF-C, Differentiation, Lymphangiogenesis

## Abstract

Endothelial progenitor cells (EPCs) play an important role in postnatal neovascularization. However, it is poorly understood whether EPCs contribute to lymphangiogenesis. Here, we assessed differentiation of a novel population of EPCs towards lymphatic endothelial cells and their lymphatic formation. CD34^+^VEGFR-3^+^ EPCs were isolated from mononuclear cells of human cord blood by fluorescence-activated cell sorting. These cells expressed CD133 and displayed the phenotype of the endothelial cells. Cell colonies appeared at 7–10 days after incubation. The cells of the colonies grew rapidly and could be repeatedly subcultured. After induction with VEGF-C for 2 weeks, CD34^+^VEGFR-3^+^ EPCs could differentiate into lymphatic endothelial cells expressing specific markers 5′-nucleotidase, LYVE-1 and Prox-1. The cells also expressed hyaluronan receptor CD44. The differentiated cells had properties of proliferation, migration and formation of lymphatic capillary-like structures in three-dimensional collagen gel and Matrigel. VEGF-C enhanced VEGFR-3 mRNA expression. After interfering with VEGFR-3 siRNA, the effects of VEGF-C were diminished. These results demonstrate that there is a population of CD34^+^VEGFR-3^+^ EPCs with lymphatic potential in human cord blood. VEGF-C/VEGFR-3 signalling pathway mediates differentiation of CD34^+^VEGFR-3^+^ EPCs towards lymphatic endothelial cells and lymphangiogenesis. Cord blood-derived CD34^+^VEGFR-3^+^ EPCs may be a reliable source in transplantation therapy for lymphatic regenerative diseases.

## Introduction

Lymphatic capillaries begin as the dilated lymphatics with the closed ends. They lack a basement membrane and do not have pericyte. Lymphatic endothelial cells are thin except for their nuclei, which protrude into the lumen. They are tethered to the surrounding bundles of collagen by anchoring filaments [1]. Lymphatic-specific markers are podoplanin, Prox-1, VEGFR-3 and lymphatic vessel endothelial hyaluronan receptor-1 (LYVE-1) [2–5]. VEGFR-3 is receptor of VEGF-C and VEGF-D, expressing specifically on the lymphatic endothelium. In contrast, vascular endothelial cells express specifically VEGFR-1, VEGFR-2 and CD31 (PACAM-1). Lymphatic vessels drain excess fluid from the extracellular spaces, absorb lipids from the intestine and transport leucocytes and antigen-presenting cells from inflammatory tissue to lymph nodes [6, 7]. Lymphatic vessels also function in removal of cell debris, dust particles and microorganisms. On the other hand, lymphatic vessels provide one of the main routes for tumour metastasis [8].

Recently, more and more attention has been focused on lymphangiogenesis. Many studies suggested that lymphangiogenesis is involved in embryonic development, wound healing, tissue regeneration and inflammation [9]. Lymphangiogenesis also occurs in tumour tissue and is related to lymphatic metastasis [10]. It is now well-established that the new lymphatic vessels form from the pre-existed vessels through proliferation, migration and tube formation of the endothelial cells. Chemokines, growth factors, adhesion molecules and extracellular matrix play important roles in lymphangiogenesis [11, 12]. In vascular biology, it is well recognized that circulating endothelial progenitor cells (EPCs) derived from bone marrow may participate in postnatal neovascularization [13, 14]. However, the contribution of EPCs to lymphangiogenesis is not as yet clearly defined. In 2003, a population of CD34^+^ endothelial precursor cells was identified in human foetal liver, cord and periphery blood. Characteristics of these cells are coexpression of CD133 and VEGFR-3 and differentiation into endothelial cells [15]. After stimulation with growth factors, only few CD34^+^ EPC-derived cells became positive for LYVE-1 [16]. Endothelial progenitor cells were detected at the growing lymphatic vessels in the cornea of mouse treated with irradiation [17] and the transplanted human kidney [18]. However, biological characteristics of EPCs with lymphatic endothelial potential remain unclear because techniques for isolation and purification of the cells and standardization of identification are greatly different. Mechanisms of EPC differentiation towards lymphatic endothelial cells and EPC behaviours in lymphangiogenesis are poorly understood.

This investigation was designed to examine biological characteristics of CD34^+^VEGFR-3^+^ EPCs isolated from human umbilical cord blood and to evaluate effects of VEGF-C/VEGFR-3 signalling pathway on differentiation of the cells towards lymphatic endothelial cells and lymphatic formation of EPC-derived cells *in vitro*. Here, we demonstrate that CD34^+^VEGFR-3^+^ EPCs exist in human umbilical cord blood and can differentiate into lymphatic endothelial cells in condition of VEGF-C induction *in vitro*. VEGF-C enhances expression of VEGFR-3 mRNA of the cells. The differentiated cells may organize into lymphatic-like capillaries in three-dimensional collagen gel and Matrigel. Our work is the first evidence that CD34^+^VEGFR-3^+^ EPCs are a novel source for lymphangiogenesis and that VEGF-C/VEGFR-3 signalling pathway plays a crucial role in differentiation and lymphangiogenesis of the cells.

## Materials and methods

### Harvest of mononuclear cells

Human umbilical cord blood (40–60 ml) was collected from the umbilical vein after spontaneous delivery or abdominal delivery. Collection of cord blood was approved by Ethical Committee of Shanghai Health Hospital for Women and Children. For anticoagulation, 2 ml of 10% ethylenediaminetetraacetic acid Na_2_ (Sigma-Aldrich, St Louis, MO, USA) was added to the blood. Ethylenediaminetetraacetic acid inhibits activation and contamination of platelets. Mononuclear cells in cord blood were isolated by density-gradient centrifugation with percoll solution (GE Healthcare, Uppsala, Sweden) at the density of 1.076. After centrifugation for 30 min. at 800 × g, the fraction of the mononuclear cells was collected. Then, the cells were washed with PBS containing 2% foetal bovine serum (FBS; Invitrogen, Carlsbad, CA, USA) in centrifugation for two times. For examining existence of CD34^+^VEGFR-3^+^ cells in mononuclear cells, the cells were incubated with mouse anti-human CD34 antibody (1:100; Miltenyi Biotec, Auburn, CA, USA) and rabbit anti-human VEGFR-3 antibody (1:100; Santa Cruz Biotechnology, Santa Cruz, CA, USA) for 50 min. at 4°C. After washing with PBS, the cells were incubated with PE-labelled goat anti-mouse IgG (1:300; Millipore, Billerica, MA, USA) and Alexa488-labelled donkey anti-rabbit IgG (1:300; Invitrogen) for 30 min. Coexpression of CD34 and VEGFR-3 on the cells was examined with an LSM510 confocal laser scanning microscope (Carl Zeiss, Oberkochen, Germany).

### Isolation and identification of EPCs

For sorting of CD34^+^VEGFR-3^+^ cells and detection of CD133^+^VEGFR-3^+^ cells, the mononuclear cells were suspended with PBS containing 1% bovine serum albumin and adjusted to 1 × 10^7^ cells/ml. After centrifugation, the cells were incubated with rabbit anti-human VEGFR-3 antibody (1:100), PE-labelled anti-human CD34 antibody and APC-labelled anti-human CD133 antibody (1:100; Miltenyi Biotec) for 50 min. at 4°C. Subsequently, the cells were incubated with Alexa488-labelled donkey anti-rabbit IgG for 30 min. at 4°C. After washing with PBS, the cells were resuspended with DMEM (Invitrogen) supplemented with 2.5% FBS [19]. CD34^+^VEGFR-3^+^ cells and CD133^+^VEGFR-3^+^ cells were analysed, and then CD34^+^VEGFR-3^+^ cells were sorted by using a Beckman MoFlo™ XDP FACS (fluorescence-activated cell sorter; Beckman Coulter, Fullerton, CA, USA) [20]. Additionally, expression of CD133 on the sorted CD34^+^VEGFR-3^+^ cells was examined with a LSM510 confocal laser scanning microscope.

To evaluate endothelial cell potential of the cells, the CD34^+^VEGFR-3^+^ cells were identified with uptake of acetylated low-density lipoprotein and Ulex Europaeus agglutinin-1 (UEA-1) binding [12]. The cells were seeded in culture dish or on coverslips and incubated for 24 hrs. Then, the cells were incubated with 10 μg/ml Dil-Ac-LDL (Biomedical Technologies, Stoughton, MA, USA) for 4 hrs at 37°C, or stained with FITC-conjugated UEA-1 (Sigma-Aldrich) for 2 hrs. The cells were examined by using a confocal laser scanning microscope.

### Induction of cell differentiation

The freshly sorted CD34^+^VEGFR-3^+^ EPCs were suspended with DMEM supplemented with 50 ng/ml VEGF-C (Sigma-Aldrich), 15% FBS, 100 U/ml penicillin and 100 μg/ml streptomycin (Beyotime, Nantong, China) and seeded onto 35 × 10 mm dishes pre-coated with fibronectin (Sigma-Aldrich) in density of 1 × 10^5^ cells/dish. The cells were incubated for 14 days at least at 37°C, 5% CO_2_, in a humidified incubator. The morphological changes of the cells were recorded with a phase-contrast microscope at days 1, 7, 10, 14 and 21 after induction respectively. The cells induced with VEGF-C for 14 days were used for all following experiments for examining lymphatic formation of the cells.

### Transmission electron microscopy

CD34^+^VEGFR-3^+^ EPCs and the differentiated cells were fixed with 2.5% glutaraldehyde at 4°C, and then post-fixed with 1% osmium tetroxide. After being dehydrated with gradient alcohol, the cells were soaked with anhydrous acetone and Spurr resin, and embedded with Spurr resin. Ultra-thin sections were gained with Reichert-ultracut E ultra-thin microtome (Leica, St. Gallen, Switzerland), and stained with 3% uranyl acetate and lead citrate [21]. The ultrastructural characteristics of the cells were examined by using a CM120 transmission electron microscope (Philips, Eindhoven, Holland).

### Immunocytochemistry

To evaluate differentiation of CD34^+^VEGFR-3^+^ EPCs towards lymphatic endothelial cell, VEGF-C-induced cells were harvested and seeded on coverslips coated with polylysine. The differentiated cells were identified with expression of lymphatic endothelium-specific markers 5′-nucleotidase (5′-Nase) [22], LYVE-1 and Prox-1, and hyaluronic receptor CD44. The cells were incubated with mouse anti-human JC815 (1:200; a kind gift from Dr. Seiji Kato, Department of Anatomy, Biology and Medicine, Oita University Faculty of Medicine, Japan) and rabbit anti-human LYVE-1 antibody (1:100; AngioBio, Del Mar, CA, USA) overnight at 4°C. After washing, the cells were incubated with FITC-labelled goat antimouse IgG (1:300; Jackson ImmunoResearch Laboratories, West Grove, PA, USA) and TRITC-labelled goat anti-rabbit IgG (1:300; Jackson) for 30 min. at 37°C. The nucleus was counterstained with 4′,6-Diamidino-2-phenylindole (DAPI, 1:1000; Sigma-Aldrich). For detecting expression of Prox-1 and CD44, the cells were incubated with rabbit anti-human Prox-1 antibody (10 μg/ml; Fitzgerald Industries International, North Acton, MA, USA) or mouse anti-human CD44 monoclonal antibody (1:100; Sigma-Aldrich) overnight at 4°C and then incubated with TRITC-labelled goat anti-rabbit IgG (1:300; Jackson) or FITC-labelled goat antimouse IgG (1:300; Jackson) for 30 min. at 37°C. The cells were viewed by using confocal laser scanning microscope.

### Quantitative real-time PCR

Total RNA was extracted from the CD34^+^VEGFR-3^+^ EPCs treated with VEGF-C at different time-points by using TRIzol reagent (Invitrogen). The sample RNA was reverse-transcribed to first-strand cDNA by using SYBR green PCR master mix (Applied Biosystems, Foster, CA, USA) according to the manufacturer's instructions. The level of VEGFR-3 mRNA was determined by quantitative real-time polymerase chain reaction (qPCR). VEGFR-3 primer sequences are: sense, 5′-CTGGCCAGAGGCACTAAGAC-3′; antisense, 5′-CAGGGTGTCCTCTGGGAATA-3′. The qPCR was performed on an ABI Prism 7700 system (Applied Biosystems) according to the manufacturer's instructions. Data were normalized to the reference gene GAPDH. RNA from the cells without VEGF-C treatment was used as a control.

### RNA interference (RNAi)

To evaluate the effect of VEGF-C/VEGFR-3 signalling pathway on lymphangiogenesis of the EPC-derived cells, the siRNA oligonucleotides of VEGFR-3 were designed and synthesized by Genepharma (Shanghai, China). Sequences of three specific siRNAs targeting human VEGFR-3 mRNA were following: VEGFR-3 siRNA no. 1, 5′-CGCUGAUGUCGGAGCUCAATT-3′; VEGFR-3 siRNA no. 2, 5′-GCUUCACCAUCGAAUCCAATT-3′; VEGFR-3 siRNA no. 3, 5′-CCGUGUGGGCUGAGUUUAATT-3′. An irrelevant siRNA with random nucleotides 5′-UUCUCCGAACGUGUCACGUTT-3′ was used for negative control. The siRNA oligonucleotide was not homologous to any sequences found in the gene bank. For screening the effective siRNA fragment, the cells were divided into four groups and grew into subconfluent monolayers. After incubation with serum-free medium for 12 hrs, the cells were transfected with Lipofectamine 2000 (Invitrogen) for 6 hrs according to the manufacturer's instructions. After the transfection medium was replaced with the medium containing VEGF-C, the cells were incubated for an additional 24 hrs [23]. Efficacy of siRNAs in interfering expression of VEGFR-3 mRNA was determined with RT-PCR analysis.

### Proliferation assay

In proliferation assay, EPC-derived cells were seeded onto the fibronectin-coated dishes at a density of 3 × 10^5^ cells/ml and incubated with the complete medium. After the cells were grown to submonolayers, the cells were washed with PBS and incubated for 24 hrs with serum-free medium. The dishes were divided into four groups, treated with vehicle (control group), 50 ng/ml VEGF-C (VEGF-C group), irrelevant siRNA and VEGF-C (RNAi control group) or VEGFR-3 siRNA and VEGF-C (RNAi group) respectively. In RNAi control and RNAi groups, the cells were incubated with transfection medium containing irrelevant siRNA or VEGFR-3 siRNA for 6 hrs before proliferation assay. After incubation for 24 hrs, the cells were harvested and counted with a haemocytometer. The experiment was repeated five times in each group.

### Transmigrating assay

In transmigrating assay, six well formats of cell culture inserts (BD Biosciences, Klin Lakes, NJ, USA) were used. The culture system is divided into upper chamber and lower chamber by microporous membrane. The diameter of the pores of the membrane is 8 μm. The cells were seeded at a density of 3 × 10^5^ cells/ml on the upper side of the membrane coated with fibronectin. The cells were divided into vehicle, VEGF-C, RNAi control and RNAi groups as described above. There are three repeated wells in each group. In RNAi groups, the cells were pre-treated with siRNA for 6 hrs. In VEGF-C, RNAi control and RNAi groups, 50 ng/ml VEGF-C was added into the lower chamber. After incubation for 12 hrs, the cells on two sides of the membrane were dried in air and then fixed with methanol for 20 min. Subsequently, the cells were stained with 10% Giemsa for 10 min. The cells migrated to the lower sides of the membrane were counted by using a microscope. The assay was repeated three times.

For scanning electron microscopy, the membrane was removed from the insert. The cells grown on the membrane were fixed with 2.5% glutaraldehyde overnight at 4°C, and then dehydrated with gradient alcohol. After going through another graded series to replace the alcohol with hexamethyldisilazane, the cells were dried in air and the specimen was coated with carbon. The morphological features of the cells migrated through the pores of the membrane were examined with a scanning electron microscope (FEI QUANTA200; Philips, DA Best, The Netherlands).

### Wounding assay

For comparing effects of VEGF-C and other growth factors on migration of the EPC-derived cells, wound assays were performed in a manner described previously [12]. Briefly, the cells in dishes were scraped at four sites with a 5-mm-wide razor blade when the cells were grown to monolayer. After washing in PBS, the cells were incubated with serum-free DMEM containing 0.1% gelatin and 50 ng/ml bFGF (basic fibroblast growth factor), 50 ng/ml VEGF or 50 ng/ml VEGF-C for 24 hrs. In RNAi group, the cells were pre-treated with VEGFR-3 siRNA for 6 hrs. After washing, the cells were stained with 10% Giemsa solution. The cells migrated from the wound edge were counted in successive 100 μm section of 300 μm wide by using an Olympus (Tokyo, Japan) microscope with an ocular grid. The maximal distance of cell migration from the wounded edge was measured. The assay was triplicated.

### Tube formation in three-dimensional collagen gel and Matrigel

To examine tube formation of EPC-derived cells, three-dimensional collagen type I gel was prepared according to the method as described previously [24]. In brief, type I collagen from rat tail (Sigma-Aldrich) was dissolved in acetic acid. Seven volumes of ice-cold collagen solution (2 mg/ml) were quickly mixed with one volume of 10× DMEM and two volumes of sodium bicarbonate (11.76 mg/ml). The pH of the solution was adjusted by addition of NaHCO_3_. The 35-mm dishes were coated in collagen solution (1 ml/dish) and allow to gel at 37°C for 10 min. Then, the cells were seeded at a density of 3 × 10^5^ cells/ml on collagen gel. When being grown to submonolayers, the cells were divided into four groups and treated with vehicle, VEGF-C, RNAi control and RNAi respectively for 2 hrs. Following removal of the medium, a layer of collagen gel on the cells was made by using the same method described as above. On this layer gel, 0.5 ml of the medium supplemented with 3% FBS was added, and then the cells were continued to be incubated for 24 hrs. The gel cultures were stained with 10% Giemsa solution, and randomly selected fields were photographed with a microscope (Olympus). Tube formation of the cells was quantitated by measuring the length and area of the tubes with a CKS400 image analyzer (Carl Zeiss). The experiment was repeated three times.

For transmission electron microscopy assay of the capillary-like structures, the collagen gel cultures were fixed overnight with 2.5% glutaraldehyde in 0.1 M sodium cacodylate buffer. After washing in the same buffer solution, the gel was gently removed from the dishes and trimmed into about 2 × 2 mm squares. Then, these squares were post-fixed with 1% osmium tetroxide in 0.1 M sodium cacodylate buffer for 1 hr, dehydrated in the graded ethanols and processed for Spurr resin embedding. Preparation of ultra-thin sections and ultrastructural examination were performed according to the methods described as above.

To identifying lymphatic capillary-like structures formed by EPC-derived cells, the tubes organized in Matrigel were examined with LYVE-1 immunostaining. Matrigel matrix (BD Biosciences) diluted in serum-free DMEM (1:1) was added to 35-mm dishes (150 μl/cm^2^) and allowed to gel at 37°C for 30 min. Endothelial progenitor cell-derived cells were seeded at a density of 3 × 10^5^ cells/ml on Matrigel and incubated in the medium containing 50 ng/ml VEGF-C for 12 hrs. The lymphatic capillary-like structures in Matrigel were identified with LYVE-1 immunostaining as above and viewed by using confocal laser scanning microscope.

### Statistical analysis

Experimental values were expressed as the mean ± SD. Data were analysed by using Student's unpaired *t*-test and one-way anova with Scheffe's post-hoc multiple-comparison analysis. A value of *P* < 0.05 was considered statistically significant and a value of *P* < 0.01 was considered very statistically significant.

## Results

### Sorting and detecting of CD34^+^VEGFR-3^+^ cells

**Figure 1:**
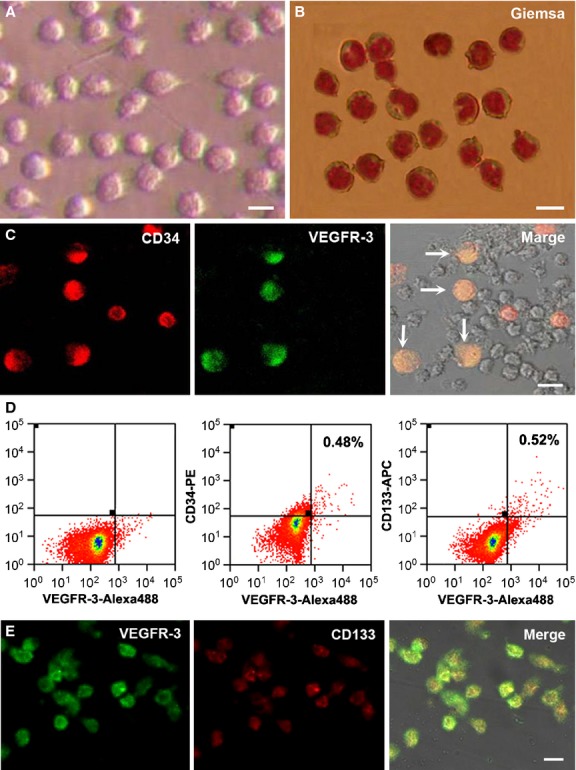
Isolation of CD34^+^VEGFR-3^+^ cells. (A) The mononuclear cells isolated from human umbilical cord blood. The cells are round and become fusiform after adhesion. (B) The mononuclear cells stained with 10% Giemsa solution. The nuclei of the cells are round-, ellipse-or horseshoe-shaped. (C) Features of CD34^+^VEGFR-3^+^ cells. In the mononuclear cells, CD34^+^VEGFR-3^+^ cells (arrows) are larger than CD34^+^VEGFR-3^−^ cells. The right panel is the merged image of the left and middle panels and phase-contrast image. Bars (A–C) represent 10 μm. (D) Phenotype of the mononuclear cells analysed by dual-colour flow cytometry. CD34^+^VEGFR-3^+^ cells and CD133^+^VEGFR-3^+^ cells present in the mononuclear cells. Percentage of the positive cells was compared with isotype control. (E) CD133 is weekly expressed on the freshly sorted CD34^+^VEGFR-3^+^ cells. Bar represents 10 μm.

The mononuclear cells isolated from human cord blood are round, having a round-, ellipse-or horseshoe-shaped nucleus (Fig. 1A and B). Immunofluorescent double staining shows that there is a population of CD34^+^VEGFR-3^+^ cells in the mononuclear cells. All VEGFR-3^+^ cells expressed CD34. The diameter of the cells coexpressing CD34 and VEGFR-3 was 10–12 μm, CD34^+^VEGFR-3^−^ cells was 7–8 μm in diameter. VEGFR-3 expression was not observed on the CD34^−^ cells (Fig. 1C). After the mononuclear cells were treated with CD34, VEGFR-3 and CD133 immunostaining, CD34^+^VEGFR-3^+^ cells and CD133^+^VEGFR-3^+^ cells were detected respectively. The result of fluorescence-activated cell sorting (FACS) reveals that CD34^+^VEGFR-3^+^ cells are 0.48 ± 0.32% and CD133^+^VEGFR-3^+^ cells are 0.52% ± 0.21% in the mononuclear cells (Fig. 1D). The freshly sorted CD34^+^VEGFR-3^+^ cells express CD133 weakly in immunostaining (Fig. 1E).

### Characterization of CD34^+^VEGFR-3^+^ cells

**Figure 2:**
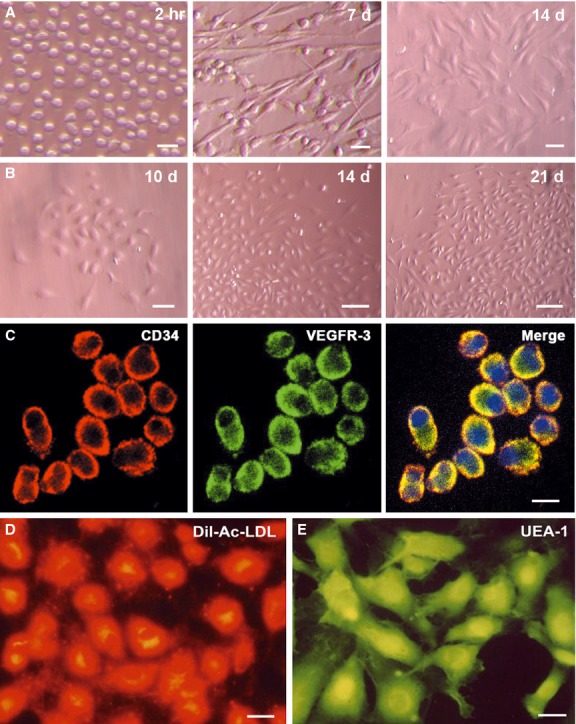
Characterization of CD34^+^VEGFR-3^+^ cells during differentiation. (A) Morphological changes of the cells during differentiation. At 2 hrs after induction with VEGF-C, the cells were round or ellipse. At day 7, the most cells became spindle-shaped. At day 14, the confluent monolayer of the cells demonstrated a typical cobblestone appearance. Bars represent 20 μm. (B) Microphotographs of a same colony. The colony enlarges gradually. Bars represent 20 μm (left panel) and 50 μm (middle and right panels) respectively. (C) Co-expression of CD34 and VEGFR-3 on the cells of the colony. Bar represents 10 μm. (D and E) Uptake of Dil-Ac-LDL and binding of UEA-1. The cells represent endothelial progenitor cell phenotype. Bars represent 10 μm.

**Figure 3:**
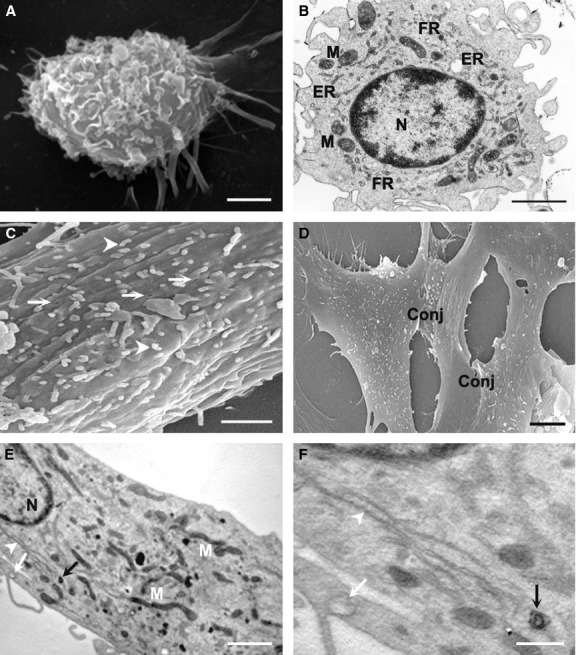
Ultrastructural characterization of CD34^+^VEGFR-3^+^ cells and the differentiated cells. The cells were treated with scanning (A, C and D) and transmission electron microscopies (B, E and F). (A) There are many microvilli on CD34^+^VEGFR-3^+^ cell. Bar represents 2 μm. (B) In CD34^+^VEGFR-3^+^ cell, the nucleus (N) is large, nuclear chromatin is rarefied. Mitochondrium (M), free ribosome (FR) and endoplasmic reticulum (ER) in the cytoplasm are rich. Bar represents 2 μm. (C and D) The cells induced with VEGF-C for 14 days. On the cell surface, there are numerous caveolae (arrows) and microvilli (arrowheads; C). Conjunctions (Conj) form between adjacent cells (D). Bars represent 1 μm (C) and 2 μm (D) respectively. (E and F) Organelles in the cells induced with VEGF-C for 14 days. There are more mitochondria (M), rough endoplasmic reticula (arrowheads) and phagocytic vesicles (white arrow) in the cytoplasm. Weibel-Palade body (black arrow) enwrapped by the membrane is observable. F is high-magnification of the selected area from E. N, nucleus. Bars represent 2 μm (E) and 0.5 μm (F) respectively.

The freshly sorted CD34^+^VEGFR-3^+^ cells were round or elliptic. At day 7 after induction with VEGF-C, the cells spread well, most of them exhibited spindle-shaped morphology. At day 14 after induction, the cells displayed polygonal shape and grew into confluent monolayer (Fig. 2A). Colonies appeared occasionally in 7–10 days, which comprised of round cells centrally with spindle-shaped cells at the periphery (Fig. 2B). Proliferation of the cells in the colonies was more rapid than other cells. The cells of the colonies expressed CD34 and VEGFR-3 (Fig. 2C). CD34^+^VEGFR-3^+^ cells were positive for Dil-labelled Ac-LDL (Dil-Ac-LDL) uptake and UEA-1 binding, matching the described EPC phenotype (Fig. 2D and E). The cells represented ultrastructural characteristics of stem/progenitor cells. There are many microvilli on the cell surface (Fig. 3A). The nucleus of the cells is large, nuclear chromatin is rarefied. The cells contain numerous mitochondria, free ribosomes and endoplasmic reticula in the cytoplasm (Fig. 3B).

### Differentiation of CD34^+^VEGFR-3^+^ cells towards lymphatic endothelial cells

**Figure 4:**
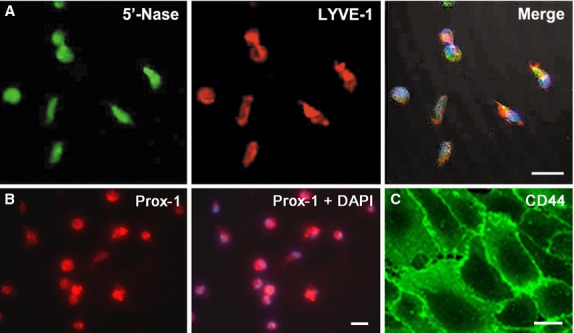
Differentiation of CD34^+^VEGFR-3^+^ cells into lymphatic endothelial cells. After induction with VEGF-C for 2 weeks, the cells express markers for lymphatic endothelial cells, 5′-Nase, LYVE-1 and Prox-1, and hyaluronan receptor CD44. (A) Expression of 5′-Nase and LYVE-1. Bar represents 20 μm. (B) Expression of Prox-1. The nucleus was counterstained with DAPI. Bar represents 10 μm. (C) Expression of CD44. Bar represents 20 μm.

With induction in VEGF-C, CD34^+^VEGFR-3^+^ cells differentiated towards lymphatic endothelial cells. The cells displayed fusiform or polygonal shape, and the confluent monolayer of the cells demonstrated a typical cobblestone appearance like endothelial cells (Fig. 2A). There were numerous caveolae and microvilli on the surface of the differentiated cells (Fig. 3C). When the cells grew into monolayer, the cells connect each other to form conjunctions, representing the characteristic of the endothelial cells (Fig. 3D). Weibel-Palade body enwrapped by the membrane could be observed (Fig. 3E and F). After induction for 2 weeks, the cells were positive for lymphatic endothelial cell makers, 5′-Nase and LYVE-1 (Fig. 4A). The cells expressed Prox-1 in the nuclei (Fig. 4B). CD44 was strongly expressed on the membrane of the cells (Fig. 4C). Without induction with VEGF-C, the cells were poor in spreading, and began to detach from the bottom of the dishes and became apoptotic at 1 week after incubation.

### Expression of VEGFR-3 mRNA and selection of VEGFR-3 siRNA

**Figure 5:**
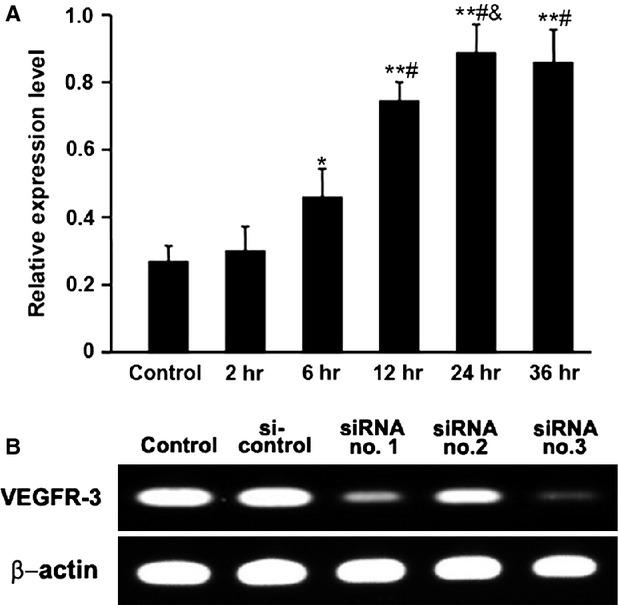
VEGFR-3 mRNA expression in CD34^+^VEGFR-3^+^ cells. (A) Changes of VEGFR-3 mRNA expression after treatment with VEGF-C. Real-time PCR analysis shows that the sorted cells express VEGFR-3 mRNA. After treatment with VEGF-C, the level of VEGFR-3 mRNA expression increases and reaches the peak at 24 hrs. *P < 0.05 *versus* control and 2 hrs; **P < 0.01 *versus* control and 2 hrs; ^#^*P* < 0.01 *versus* 6 hrs; ^&^*P* < 0.05 *versus* 12 hrs. (B) Screening of the effective VEGFR-3 siRNA. In silencing VEGFR-3 mRNA expression, VEGFR-3 siRNA no.3 is more effective than siRNA no.1 and siRNA no.2.

The result of real-time PCR showed that CD34^+^VEGFR-3^+^ EPCs sorted from the mononuclear cells of human cord blood expressed VEGFR-3 mRNA. After treatment with VEGF-C, the level of VEGFR-3 mRNA expression in the cells increased and reached the peak at 24 hrs (Fig. 5A). For selecting the effective VEGFR-3 siRNA, EPCs were transfected with siRNAs, and then the level of VEGFR-3 mRNA expression was analysed with RT-PCR. Of the three siRNAs, VEGFR-3 siRNA no.3 is most effective in suppressing VEGFR-3 mRNA expression (Fig. 5B). Therefore, VEGFR-3 siRNA no.3 was used in the following experiments of lymphatic formation.

### Proliferation and migration of EPC-derived cells

**Figure 6:**
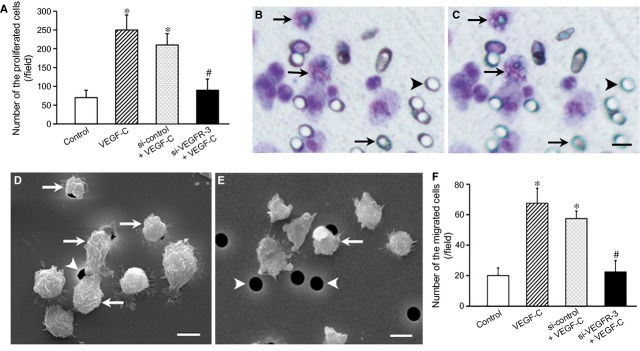
Proliferation and transmigration of endothelial progenitor cells (EPC)-derived cells. EPCs were induced with VEGF-C for 14 days before accessing their capacity of proliferation and transmigration. (A) Effect of VEGF-C on proliferation of EPC-derived cells. EPC-derived cells were seeded onto the fibronectin-coated dishes. VEGF-C increases proliferation of the cells. After the cells are treated with VEGFR-3 siRNA, the effect of VEGF-C is inhibited. *P < 0.01 *versus* control; ^#^*P* < 0.01 *versus*VEGF-C group and si-control + VEGF-C group. (B and C) Transmigration of EPC-derived cells. The cells were seeded on fibronectin-coated membrane of cell culture inserts. After stimulation of VEGF-C in the lower chamber, the cells in the upper side of the membrane (B) migrate to the lower side of the membrane (C) through pores (arrowheads). Arrows indicate the migrated cells. Giemsa staining. Bars represent 10 μm. (D and E) Scanning electron microphotographs of the cells on the membrane. There are more migrating cells (arrows) in VEGF-C group (D) compared with RNAi group (E). Arrowheads indicate pores. Bars represent 10 μm. (F) Effect of VEGF-C on transmigration of EPC-derived cells. VEGF-C promotes migration of the cells into lower chamber through pores of the membrane. After treatment with VEGFR-3 siRNA, effect of VEGF-C on cell transmigration decreases. *P < 0.01 *versus* the control group; ^#^*P* < 0.01 *versus*VEGF-C group and si-control + VEGF-C group.

**Figure 7:**
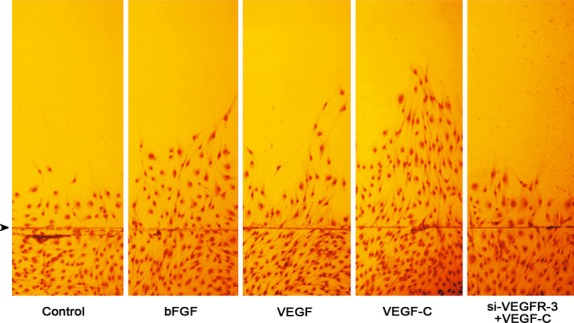
Effects of bFGF, VEGF and VEGF-C on migration of endothelial progenitor cell-derived cells. After wounding, the cells derived from CD34^+^VEGFR-3^+^ cells migrate from the monolayer side to the scraped side. The cells were treated with bFGF, VEGF or VEGF-C (50 ng/ml) for 24 hrs. The number of cells migrated to the scraped side and maximal distance of cell migration in VEGF-C group are more than that in bFGF and VEGF groups. After pre-treatment with VEGFR-3 siRNA, the effect of VEGF-C on cell migration is inhibited. Arrowhead indicates the wound edge.

After induction with VEGF-C for 24 hrs, the number of the cells increased significantly compared with the control group. When the cells were transfected with VEGFR-3 siRNA, the number of the proliferated cells decreased (Fig. 6A). In transmigration experiment, VEGF-C stimulated the cells to migrate from the upper side to the lower side of the membrane through pores of the membrane in cell culture insert (Fig. 6 B–E). The number of the transmigrated cells in VEGF-C group was greater than that in the control group. When the cells were treated with VEGFR-3 siRNA, the effect of VEGF-C on transmigration of the cells was inhibited (Fig. 6F). After wounding, the cells moved from the monolayer side into the wounded area. The number of migrated cells and the maximal distance of cell migration in VEGF-C group are greater significantly than that in bFGF and VEGF groups. In VEGFR-3 siRNA group, migration of the cells was suppressed (Fig 7, Table 1).

**Table 1:** Effects of bFGF, VEGF and VEGF-C on migration of the EPC-derived cells

Groups	Cell numbers	Maximal distance (μm)
Control	14 ± 3	239 ± 36
bFGF	36 ± 8*	398 ± 28*
VEGF	30 ± 6*	386 ± 42*
VEGF-C	48 ± 10*, †	578 ± 48*, †
RNAi	16 ± 5#	242 ± 39#

The values are mean ± SD.

* *P* < 0.01 *versus* control group

† *P* < 0.01 *versus* bFGF and VEGF groups

# *P* < 0.01 *versus* VEGF-C group. *n* = 16.

### Lymphatic formation of EPC-derived cells in three-dimensional gels

**Figure 8:**
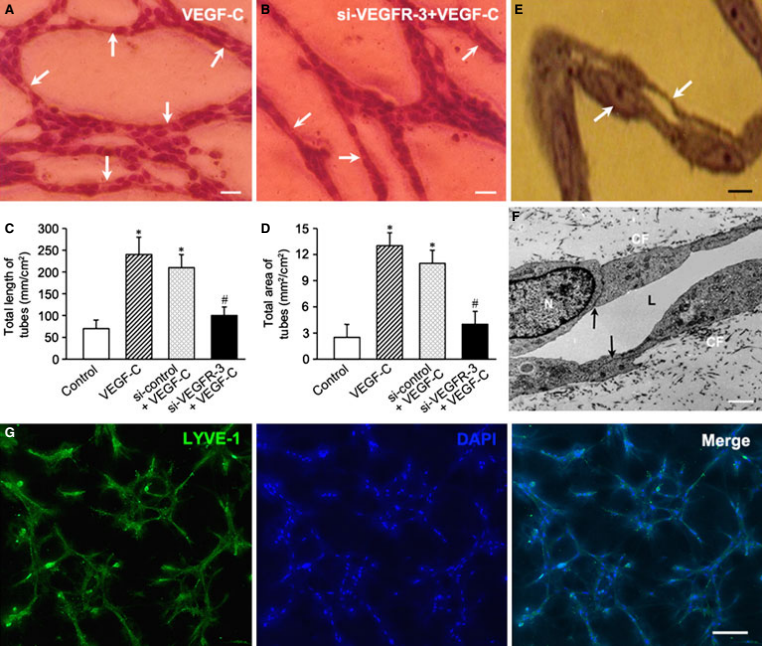
Capillary-like structures formed by endothelial progenitor cells (EPC)-derived cells. When the cells on collagen gel were grown to submonolayer, the cells were treated with VEGF-C for 2 hrs. Then, upper layer of collagen gel on the cells was made. The cells were continued to be incubated for 24 hrs. (A and B) Microphotographs of capillary-like structures formed by EPC-derived cells. VEGF-C stimulates the cells to organize into capillary-like structures (A). After treatment with VEGFR-3 siRNA, the cells cannot form tubes well (B). Arrows indicate the tubes. Giemsa staining. Bars represent 20 μm. (C and D) Statistical analysis of length and area of the tubes. Length and area of the tubes in VEGF-C group are greater than those in the control and VEGFR-3 siRNA groups. *P < 0.01 *versus* the control group; ^#^*P* < 0.01 *versus*VEGF-C group and si-control/VEGF-C group. (E) Microphotograph of capillary-like structures in semi-thin section. The tube displayed structures of capillaries with a lumen. The wall of the tube is very thin. Bar represents 5 μm. (F) Transmission electron microphotograph of capillary-like structures. The adjacent cells were extensively overlapping. There were tight junctions between the adjacent cells (arrows). The basal lamina is absent in the tube. The lumen (L) of the tube was irregular. CF, collagen fibres in the gel. Bar represents 1 μm. (G) LYVE-1 expression of capillary-like structures formed by EPC-derived cells in Matrigel. The cells were incubated in the medium containing 50 ng/ml VEGF-C for 12 hrs. The capillary-like structures in Matrigel were identified with LYVE-1 immunostaining. Bars represent 100 μm.

Endothelial progenitor cell-derived cells could organize into capillary-like tubes in the three-dimensional collagen gel. In VEGF-C group, the tubes connected each other and formed a network (Fig. 8A). After treatment with VEGFR-3 siRNA, the formation of capillary-like tubes by the cells was inhibited (Fig. 8B). Length and area of the tubes in VEGF-C group were greater than those in the control group. After suppression of VEGFR-3 expression with siRNA, tube formation of the cells was inhibited significantly (Fig. 8C and D). In the semi-thin sections, the tubes displayed structures of capillaries with a lumen (Fig. 8E). In the ultra-thin sections, the capillary-like structure was composed of two or three cells. The adjacent cells were extensively overlapping. There were tight junctions between the adjacent cells (Fig. 8F). The lumen of the tube was irregular. In some tubes, there was remnant collagen, which had not been degraded.

In condition of treatment with VEGF-C, EPC-derived cells could form capillary-like structures within Matrigel. The structures were positive for LYVE-1 immunostaining.

## Discussion

In the present study, we demonstrate that CD34^+^VEGFR-3^+^ cells sorted from mononuclear cells of human umbilical cord blood also express CD133 weakly. CD34^+^VEGFR-3^+^ EPCs have a potential to differentiate towards lymphatic endothelial cells and then form lymphatic capillaries in three-dimensional gels. In 1997, a population of CD34^+^VEGFR-2^+^ EPCs was isolated firstly from human peripheral blood. These cells differentiated into endothelial cells expressing VEGFR-2 and CD31 *in vitro* and incorporated into the blood capillaries in ischaemic tissue [25]. CD34^+^CD133^+^VEGFR-2^+^ cells constitute a phenotypically and functionally distinct population of circulating EPCs that play a role in neo-angiogenesis [26]. CD34 is a haematopoietic stem-cell marker, while CD133 (originally called AC133) is a haematopoietic stem-/progenitor-cell marker. Many lines of evidence show that VEGFR-3 expresses on lymphatic vessel sprouting from embryonic vein as well as postnatal lymphatic endothelium specifically [4, 5]. VEGFR-3 may be regarded as a key marker of lymphatic progenitors. Unlike studies of other groups [15, 16], this study investigated potential of differentiation towards lymphatic endothelial cells and lymphatic formation of EPCs by using the sorted CD34^+^VEGFR-3^+^ cells. The cells have endothelial cell potential, including uptake of Dil-Ac-LDL and binding of UEA-1. In flow cytometric analysis of EPCs that are capable of differentiating towards vascular endothelial cells, CD34 and VEGFR-2 are commonly used [27, 28]. Comparing CD34^+^CD133^+^VEGFR-2^+^ EPCs [26], CD34^+^VEGFR-3^+^ EPCs identified in this study may differentiate into lymphatic endothelial cells and then undergo lymphatic formation. In view of differences in the surface markers, differentiation tendency and biological function, we suggest that there are two populations of EPCs in cord blood, lymphatic endothelial progenitor cells (LEPCs) and vascular endothelial progenitor cells (VEPCs). Whether VEGFR-2^+^ EPCs and other phenotypes of EPCs may contribute to lymphangiogenesis remains unknown. Although transplantation of marrow-derived VEGFR-2^+^ EPCs resulted in cell incorporation into the newly formed lymphatic vessels [15], effect of VEGFR-2^+^ EPCs to lymphangiogenesis needs to be elucidated. The result of cell transplantation suggested that haematopoietic stem cells can incorporate into normal and tumour lymphatics [29]. Because only few specific marks are available for identifying LEPCs *in vivo* at present, identification for LEPCs should be careful *in vivo,* although GFP labelling is useful in cell-transplantation experiment. For example, lymphatic endothelial cells express CD34 as well as VEGFR-3 in some cases [4, 30]. Macrophages and dendritic cells expressing VEGFR-3 in the inflamed tissue [31, 32], possibly mistaking for LEPCs, may migrate into lymphatic capillaries.

Umbilical cord blood is a rich and ethical EPC source for treatment of vascular diseases [33]. Recently, differentiation of EPCs derived from human cord blood has been investigated intensely [20, 34, 35]. Cord blood contains more EPCs than adult peripheral blood [36]. We found that number of LEPCs in cord blood is about 10 times of that in peripheral blood (data not shown). Endothelial progenitor cells derived from cord blood have higher colony-forming and proliferative potential than that from adult peripheral blood [26, 37]. In this study, colonies formed by CD34^+^VEGFR-3^+^ EPCs appear occasionally in 7–10 days after induction with VEGF-C. Proliferation of the cells in the colonies was rapid. CD34^+^VEGFR-3^+^ EPCs in cord blood may represent a novel source of cells for lymphangiogenic therapies. Although LEPCs derived from cord blood are rare for transplantation, the cells can be expanded under VEGF-C induction *in vitro*, especially colony-forming cells. This strategy enables transplantation with enough number of EPCs for treating some diseases linked to dysfunction of LEPCs and lymphatic endothelial cells.

Our data show that VEGF-C/VEGFR-3 signalling pathway mediates differentiation of CD34^+^VEGFR-3^+^ EPCs into lymphatic endothelial cells. VEGF-C binds with VEGFR-3 specifically as well as enhances VEGFR-3 mRNA expression of the cells. The differentiated cells expressed markers for lymphatic endothelial cells, 5′-Nase, LYVE-1 and Prox-1. In addition, the cells express hyaluronan receptor CD44 strongly. One of the functions of the lymphatic vessels is transporting hyaluronan from tissues. Expression of hyaluronan receptors LYVE-1 and CD44 on the lymphatic endothelial cells differentiated from EPCs is probably implicated in transportation of hyaluronan. It is well-established that VEGF-C is essential for formation of the lymphatic vessel sprouting from the embryonic vein [38]. Moreover, VEGF-C is required for development of lymphatic vessel in embryoid body formed from embryonic stem cells [39, 40]. With induction of VEGF-C or lymph-inductive media, multipotent mesenchymal stem cells are capable of expressing a lymphatic phenotype [41]. On the other hand, VEGFR-3 expression becomes largely restricted to the lymphatic vessels during later embryonic development [4, 5]. Overexpression of VEGFR-3 into embryonic stem cell-derived vascular progenitor cells stimulates LYVE-1 expression [42]. The targeted inactivation of the gene encoding VEGFR-3 results in failure in formation of initial lymphatic system [43]. These lines of evidence indicate that VEGF-C induces differentiation of lymphatic stem/progenitor cells towards endothelial cells *via* VEGFR-3 signalling pathway. Therefore, this study suggests that VEGF-C is a pivotal cytokine for differentiation of VEGFR-3^+^ EPCs into lymphatic endothelial cells.

The findings in this study provide the first evidence for lymphangiogenesis of CD34^+^VEGFR-3^+^ EPC-derived cells in the extracellular matrix *in vitro*. Furthermore, our results demonstrate that endothelial cells derived from CD34^+^VEGFR-3^+^ EPCs represent similar behaviours to native lymphatic endothelial cells in lymphangiogenesis *in vitro* [12]. VEGF-C facilitates EPC-derived cells to proliferate, migrate and form lymphatic capillary-like tubes *via* VEGFR-3 signalling. In wounding assay, effect of VEGF-C on migration of the cells is greater than that of bFGF and VEGF. Many lines of evidence show that VEGF-C expression of inflammatory cells and tumour cells increases in some inflammatory and malignant diseases [44–45]. Up-regulation of VEGF-C expression is related to lymphangiogenesis [47, 48]. Therefore, VEGF-C produced in the local tissue may induce differentiation and lymphangiogenesis of LEPCs. Our observations, together with the findings of other research groups [17, 18, 49], suppose the processes of postnatal LEPC-induced lymphvasculogenesis, including mobilization from bone marrow to peripheral blood, transmigration through capillaries, homing to the local tissue, differentiation towards lymphatic endothelial cells and participation in formation of new lymphatic vessels. Postnatal neovascularization in the physiological or pathological events is consistent with neovessel formation contributed by angiogenesis and vasculogenesis at the various rates between their two mechanisms [50, 51]. Similarly, postnatal generation of new lymphatic vessels is contributed by lymphangiogenesis from the pre-existed vessels through remodelling and expansion [1] or/and by lymphvasculogenesis through LEPC incorporation. In contrast to VEPCs, LEPCs migrating from blood capillary have to enter the local tissue and then incorporate to lymphatic capillary. Because lymphatic vessels function in transporting inflammatory and antigen-presenting cells from extravascular spaces to lymph nodes, fate of LEPCs on the wall of lymphatic capillaries requires farther investigation. The transplanted VEGFR-3^+^ or VEGFR-2^+^ cells were observed in the newly formed lymphatic vessels [17]. With *in situ* hybridization of the Y chromosome, recipient-derived LEPCs were detected in new formed lymphatic vessels in human renal transplant [18]. In tumour lymphangiogenesis, bone marrow-derived EPCs did not significantly contribute to the formation of the tumour lymphatic vessels [52], while EPCs were present in peritumoral lymphatic vessels of a fibrosarcoma [17]. One of interpretations for these results might be that EPCs are not easy to migrate into intratumoral lymphatic vessels because these vessels are compressed [53, 54].

This study demonstrates that CD34^+^VEGFR-3^+^ EPCs can differentiate into lymphatic endothelial cells and subsequently organize into lymphatic capillary-like structures in three-dimensional matrix *via* VEGF-C/VEGFR-3 signalling pathway. Therefore, VEGF-C or VEGFR-3 implies as a potential target for inhibiting EPC-induced lymphangiogensis to suppress graft rejection and tumour metastasis.
